# The onset inflammatory parameters and the Cobb angle in adolescent idiopathic scoliosis: a case-control study

**DOI:** 10.3325/cmj.2025.66.352

**Published:** 2025-10

**Authors:** Gülseren Demir Karakılıç, Esra Şahingöz Bakırcı

**Affiliations:** 1 Yozgat City Hospital, Yozgat, Turkey; 2 Department of Physical Medicine and Rehabilitation, Yozgat Bozok University, Yozgat, Turkey

## Abstract

**Aim:**

To investigate the association between early inflammatory markers: neutrophil/lymphocyte ratio (NLR), monocyte/lymphocyte ratio (MLR), platelet/lymphocyte ratio (PLR), and C-reactive protein/albumin ratio (CAR) and the Cobb angle in adolescent idiopathic scoliosis (AIS).

**Methods:**

This multicenter case-control study (2017-2024) enrolled 419 patients with AIS (Cobb angle >10°) and 381 age- and sex-matched controls (Cobb angle 0°). We collected demographic data, curve characteristics, and laboratory parameters (white blood cells [WBC], NLR, MLR, PLR, and CAR) at the time of initial diagnosis, before treatment initiation or infection. The Cobb angle was measured by two independent radiologists blinded to clinical data.

**Results:**

Compared with controls, AIS patients had significantly higher WBC, neutrophil, lymphocyte, and monocyte counts, CRP, NLR, and CAR, and lower platelets, PLR, and calcium levels (all *P* < 0.05). CAR and NLR values increased with greater Cobb angle. In contrast, mean platelet volume did not differ between the groups.

**Conclusion:**

This is the first study to evaluate CAR in AIS, demonstrating its significant association with the Cobb angle, alongside NLR and MLR. As inexpensive and readily available markers, CAR and NLR may help in diagnosing AIS, but their role in risk stratification and disease monitoring requires confirmation in future prospective studies.

Adolescent idiopathic scoliosis (AIS) is a progressive three-dimensional spinal deformity marked by one or more lateral curvatures with vertebral rotation, defined as a Cobb angle ≥10° (1). Globally, scoliosis has a prevalence of 1%-4% and an incidence of 2.3% (3.1% in women, 1.5% in men) (2). By 2050, the prevalence of scoliosis in skeletally mature populations may nearly double, posing a considerable burden on health care systems (3).

Despite extensive study, the etiology of about 80% of scoliosis cases remains idiopathic. AIS typically develops gradually during growth, with curve progression often paralleling skeletal maturation. A multifactorial pathogenesis is widely accepted, involving genetic predisposition, connective tissue dysregulation, musculoskeletal imbalances, endocrine factors (eg, melatonin), neuromuscular control, vestibular abnormalities, and platelet/vascular microstructure. Recent reviews highlight genetic contributions and polygenic risk models as promising directions for understanding AIS susceptibility and progression. In addition, recent comprehensive reviews emphasize the interplay of developmental mechanisms, biomechanical complexities, and novel molecular insights in AIS pathogenesis (4,5).

Mechanical loading and repetitive micromovements of the spine may provoke microdamage and remodeling, triggering a chronic low-grade inflammatory response (6). Inflammation, even at subclinical levels, may influence vertebral growth modulation, bone turnover, and paraspinal soft tissue remodeling. Therefore, identifying systemic inflammatory markers at early diagnosis is of interest for prognostication in AIS (7).

Standard leukocyte subsets (neutrophils, lymphocytes, monocytes) have long been used as basic inflammatory indicators, but composite ratios such as neutrophil/lymphocyte ratio (NLR), monocyte/lymphocyte ratio (MLR), and platelet/lymphocyte ratio (PLR) more sensitively reflect systemic inflammation in musculoskeletal conditions (8). More recently, the C-reactive protein/albumin ratio (CAR) has gained attention as a novel biomarker in oncology, cardiovascular disease, and autoimmune disorders, though it has not yet been evaluated in scoliosis (9-12).

Only a few studies have addressed the links between inflammation and curve severity in AIS. One study compared adolescents with a Cobb angle ≥10°, those at risk of AIS (Cobb <10°), and healthy controls. Mean platelet volume (MPV) was elevated in the AIS group; NLR was higher as well, though not significantly (13). In another retrospective cohort of 184 patients with AIS, PLR was significantly correlated with the Cobb angle and reduced bone mineral density (14). Recent studies have also explored novel biomarker and proteomic signatures related to curve severity, oxidative stress, and extracellular matrix remodeling in AIS (15-17). Given limited and conflicting evidence on inflammatory biomarkers and the absence of data on CAR in AIS, the present study aimed to systematically assess the relationship between onset inflammatory parameters (NLR, MLR, PLR, CAR) and the Cobb angle in patients with AIS at initial diagnosis, compared with matched controls with a Cobb angle of 0°.

## PATIENTS AND METHODS

### Study design

This multicenter, case-control study was conducted between January 2017 and January 2024 at two Physical Medicine and Rehabilitation outpatient clinics: Yozgat City Hospital and Yozgat Bozok University, Faculty of Medicine, both located in Yozgat, Turkey. The study was approved by the Non-invasive Clinical Research Ethics Committee of Bozok University.

### Patients

Data on all adolescents aged 10-18 years who underwent standing posteroanterior and lateral whole-spine radiography during the study period were retrospectively screened. The patient group included individuals diagnosed with AIS with a Cobb angle >10° on radiography who had complete blood count and biochemical parameters measured at the time of the first diagnosis, before initiation of any treatment. The control group included adolescents of similar age and sex with a Cobb angle of 0°, confirmed by whole-spine radiography, who had routine blood tests performed during health check-ups. All controls underwent whole-spine radiography to exclude scoliosis, and electronic medical records were reviewed to rule out chronic inflammatory disease or relevant medication use.

Exclusion criteria (applied to both groups) were a history of infectious disease in the last month, active infection, use of antibiotics/antivirals, known rheumatological or inflammatory disease, chronic systemic illness, and the use of anti-inflammatory or immunosuppressive medications. All exclusions were based on electronic medical records available at the time of blood sampling.

### Data collection

Demographic data (age, sex), curve characteristics (location, direction, and the Cobb angle), and laboratory results were retrieved. Laboratory values included hemoglobin (g/dL), white blood cell (WBC, ×10^3^/μL), neutrophil ( × 10^3^/μL), lymphocyte ( × 10^3^/μL), monocyte ( × 10^3^/μL), and platelet count ( × 10^3^/μL), MPV (fL), creatinine (mg/dL), albumin (g/dL), calcium (mg/dL), phosphorus (mg/dL), and CRP (mg/dL). Inflammatory indices were calculated as follows: NLR: neutrophils/lymphocytes; MLR: monocytes/lymphocytes; PLR: platelets/lymphocytes; and CAR: CRP/albumin. All measurements were obtained at the initial hospital admission. PLR was expressed as a ratio without a unit.

### The Cobb angle measurement

The Cobb angle was measured using the standard method on standing thoracolumbar radiographs. All measurements were independently performed by two specialists in physical medicine and rehabilitation blinded to clinical and laboratory data. Inter-observer agreement was >90%. Discrepancies were resolved by consensus. Patients with a Cobb angle >10° were classified as having scoliosis, while those with a Cobb angle of 0° served as controls.

### Statistical analysis

Continuous variables were expressed as mean ± standard deviation or median (interquartile range), and categorical variables as number and percentage. Normality of distribution was assessed with the Kolmogorov-Smirnov test. Between-group comparisons were made with an independent-samples *t* test or a Mann-Whitney U-test, as appropriate. Differences in categorical variables were assessed with a Pearson χ^2^ test or Fisher exact test. Comparisons among more than two groups were made with a Kruskal-Wallis test followed by a *post-hoc* Dunn’s test. A two-tailed *P* value <0.05 was considered statistically significant. The analysis was performed with SPSS version 26.0 (IBM Corp., Armonk, NY, USA).

## RESULTS

A total of 800 adolescents were included: 419 patients with AIS and 381 healthy controls with a Cobb angle of 0°. The groups were comparable in terms of mean age and sex distribution (Table 1). Most of our patients had predominantly thoracolumbar localization of AIS (85.7%) (Table 2).

**Table 1:** Demographic and baseline laboratory values of adolescent idiopathic scoliosis (AIS) patients and controls*

Variable	AIS group (n = 419)	Control group (n = 381)	Reference range	p
Age (years)	14.0 ± 2.0	14.0 ± 2.0	-	0.540
Sex (F/M)	257/162	234/147	-	1.000
White blood cells ( × 10^3^/μL)	8.6 (3.4)	6.3 (0.7)	3.8-11.0	<0.001
Hemoglobin (g/dL)	14.1 (1.5)	14.0 (1.6)	11.0-16.0	0.510
Mean corpuscular volume (fL)	83.7 (6.5)	85.0 (5.8)	76-96	<0.001
Platelet count ( × 10^3^/μL)	272 (86)	282 (82)	150-400	0.010
Neutrophil count ( × 10^3^/μL)	5.2 (3.5)	2.8 (1.5)	2.0-7.0	<0.001
Lymphocyte count ( × 10^3^/μL)	2.2 (1.2)	1.9 (0.7)	1.3-3.5	<0.001
Monocyte count ( × 10^3^/μL)	0.60 (0.3)	0.50 (0.2)	0.2-0.8	<0.001
C-reactive protein (mg/dL)	1.0 (0.7)	0.3 (0.1)	<0.8	<0.001
Albumin (g/dL)	4.4 (0.5)	4.5 (0.6)	3.5-5.2	<0.001
Calcium (mg/dL)	9.7 (0.4)	9.8 (0.5)	8.8-10.6	<0.001
Phosphorus (mg/dL)	4.0 (0.2)	4.0 (0.1)	2.5-4.5	0.026

*Data are presented as mean (standard deviation).

**Table 2:** Curve characteristics in adolescent idiopathic scoliosis (AIS) patients

Characteristic	No. of AIS patients (n = 419)	%
Curve location		
thoracolumbar	359	85.7
thoracic	44	10.5
lumbar	16	3.8
Side		
right	144	34.4
left	275	65.6
Cobb angle		
11-20°	354	84.5
>20°	65	15.5

**Figure 1:**
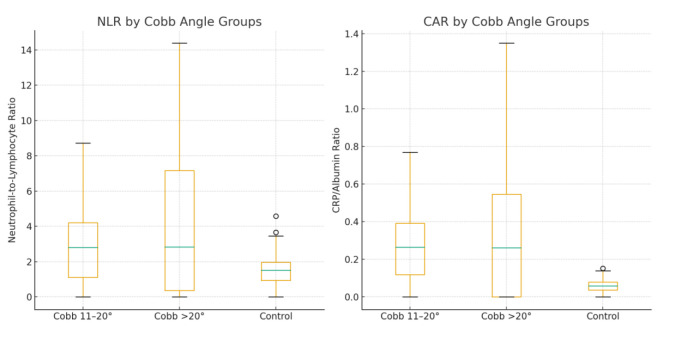
Neutrophil-to-lymphocyte ratio (NLR) and C-reactive protein/albumin ratio (CAR) in controls and two groups of patients with adolescent idiopathic scoliosis

Compared with controls, the scoliosis group had significantly higher WBC, neutrophils, lymphocytes, monocytes, CRP, NLR, CAR, and phosphorus (*P* < 0.01 for all). In contrast, it had lower red cell distribution, MCV, platelet count, PLR, and calcium levels (*P* < 0.01). MPV values were similar between the groups (*P* = 0.287). The proportions of abnormal categories (low, normal, high) for WBC, hemoglobin, MCV, neutrophils, lymphocytes, monocytes, CRP, and calcium also differed significantly between the groups (Table 1). NLR and CAR progressively increased with curve severity (Figure 1).

When stratified by Cobb angle severity (11-20° and >20°), both scoliosis subgroups demonstrated higher neutrophil, lymphocyte, and monocyte counts than controls (*P* < 0.001 for most comparisons). They also had significantly lower platelet counts (*P* < 0.01). No significant differences were found in MPV (Table 3). Furthermore, both scoliosis subgroups had significantly higher NLR, MLR, PLR, and CAR (*P* < 0.001 for NLR, PLR, CAR; *P* < 0.01 for MLR). Among scoliosis patients, CAR and CRP values were significantly higher in patients with a Cobb angle >20° compared with those with the angle of 11-20° (*P* < 0.001 for all). The two scoliosis subgroups did not significantly differ in NLR, MLR, or PLR (Table 3).

**Table 3:** Inflammatory indices according to Cobb angle severity*

Variable	Cobb = 0° (n = 381)	Cobb 11-20° (n = 354)	Cobb >20° (n = 65)	p
Neutrophil ( × 10^3^/μL)	2.8 (1.5)	5.85 (3.1)	5.95 (6.7)	<0.001
Lymphocyte ( × 10^3^/μL)	1.9 (0.7)	2.4 (1.2)	2.0 (1.1)	<0.001
Monocyte ( × 10^3^/μL)	0.50 (0.2)	0.68 (0.3)	0.65 (0.4)	<0.001
Platelet ( × 10^3^/μL)	282 (82)	279 (83.7)	281 (83.7)	0.010
**Mean platelet volume** (fL)	8.2 (1.0)	8.1 (1.3)	7.9 (1.7)	0.287
**Neutrophil-to-lymphocyte ratio**	1.46 (0.81)	2.35 (2.49)	2.85 (4.51)	<0.001
**Monocyte-to-lymphocyte ratio**	0.26 (0.12)	0.28 (0.17)	0.28 (0.27)	0.010
**Platelet-to-lymphocyte ratio**	0.14 (0.07)	0.12 (0.07)	0.14 (0.09)	<0.001
C-reactive protein (CRP)-to-albumin ratio	0.06 (0.03)	0.25 (0.20)	0.28 (0.52)	<0.001
CRP (mg/dL)	0.3 (0.1)	1.1 (0.8)	1.2 (2.1)	<0.001

## DISCUSSION

In this study, patients with AIS had significantly higher WBC, neutrophil, lymphocyte, and monocyte counts; CRP, NLR, and CAR; and lower platelets, PLR, and calcium. Importantly, CAR and NLR values were positively associated with increasing Cobb angle severity, a finding that suggests a possible link between systemic inflammation and curve progression. MPV did not differ between the groups. CAR and NLR progressively increased with increasing curve magnitude. Nevertheless, AIS etiology remains multifactorial, and inflammation should be considered as one of several interacting mechanisms alongside genetic, endocrine, neuromuscular, and biomechanical factors (18,19).

Female predominance (61.3%) and mean age (14 ± 2 years) observed in this study are consistent with other epidemiological studies (2,3,20). Our patients predominantly had thoracolumbar localization (85.7%), whereas other reports mostly observed thoracic curves (2,21). These discrepancies may reflect regional or methodological differences in patient recruitment and curve classification. Recent comprehensive reviews have also emphasized that AIS is a multifactorial disease, with genetic, biomechanical, and endocrine contributions remaining central to the current etiological models. Recent bibliometric and review studies have highlighted the growing role of genetic and epigenetic mechanisms in AIS pathogenesis, as well as environmental and nutritional cofactors that may interact with inflammatory pathways (18,19).

One of the factors implicated in AIS pathogenesis is calcium homeostasis. Some studies have reported that reduced calcium and poor mineralization in patients with AIS may contribute to skeletal fragility during pubertal growth (22,23). However, Catan et al (24) and Bala and Bala (14) found no significant differences in calcium levels between AIS patients and controls. In our study, calcium levels were significantly lower in patients with AIS, but remained within reference ranges in over 95% of both groups, which indicates their limited clinical relevance. Thus, calcium alone may not be a reliable biomarker for AIS. Consistent with this, recent meta-analyses suggest that deficiencies in vitamin D and alterations in body composition (lean mass) may contribute to curve susceptibility, underscoring the multifactorial metabolic component of AIS (25,26). Most parameters, while significantly different between the groups, remained within laboratory reference ranges, which may limit their direct clinical importance.

Our results regarding leukocyte counts and ratios complement previous findings. In contrast to two previous studies (13,27), we observed significantly higher WBC, neutrophil, monocyte, and lymphocyte counts in the scoliosis group. Both previous studies (13,27) involved patients with a Cobb angle of 0-10° as the control group. Our study was comparable to these studies in terms of female/male ratio and average age. The discrepancy could be explained by a high number of patients in both the scoliosis and patient group, a higher number of participants with a Cobb angle from 10-20° in the scoliosis group (284 patients, 67.78%), and the entire control group consisting of participants with a Cobb angle of 0°.

Inflammatory indices have been explored in several musculoskeletal and chronic pain conditions, which offers a useful framework for interpreting our results. In a retrospective study of 237 individuals with primary hip osteoarthritis, PLR was an independent predictor of severe disease. This finding suggests that decreased platelet activity relative to lymphocytes may reflect chronic inflammatory burden in degenerative joint conditions (28). Similarly, NLR was significantly elevated in patients with fibromyalgia compared with controls, a finding that supports the role of this ratio as a systemic inflammatory biomarker even in non-structural musculoskeletal disorders (29). In AIS, evidence has been less consistent. For example, Çelik et al (13) divided 292 adolescents into three groups: those with a Cobb angle ≥10°, those with a Cobb angle <10° but considered at risk, and healthy controls without scoliosis. Although NLR was higher in the scoliosis group, this difference did not reach statistical significance. In contrast, Bala and Bala (14) stratified 184 adolescents into three groups based on the Cobb angle (21-35°, 10-20°, and <10°). They found both NLR and MLR to be similar across groups, but PLR was significantly lower in patients with a greater Cobb angle. This suggests a possible link between platelet indices and curve severity. Our findings are partially consistent with these reports. Similar to Bala et Bala (14), we observed significantly lower PLR values in patients with AIS compared with controls, particularly in those with a Cobb angle from 11-20°. This pattern was also comparable to the reduction in PLR seen in severe hip osteoarthritis (24), indicating that lower PLR may represent a more general marker of musculoskeletal disease activity rather than being specific to AIS. Conversely, in our study, NLR and MLR were significantly higher in patients with a greater Cobb angle, which aligns with results from fibromyalgia studies (25) but diverges from scoliosis-specific reports (13,14). These discrepancies may be explained by methodological differences. In our study, the sample size was larger, the control group was strictly defined by a Cobb angle of 0°, and patients with recent infection or inflammatory comorbidities were excluded. Furthermore, our scoliosis group consisted of many patients with a Cobb angle from 11-20° (67.8%), which may have increased statistical power to detect subtle differences in NLR and MLR. Taken together, these findings suggest that while PLR appears consistently reduced in AIS and other musculoskeletal disorders, NLR and MLR show variable associations that may depend on cohort size, control group definition, and methodological rigor. Importantly, an increase in NLR and MLR in our study supports the hypothesis of a systemic low-grade inflammatory milieu in patients with AIS with higher curve severity.

To the best of our knowledge, this is the first study to examine CAR in AIS. Elevated CAR has been linked to disease activity in rheumatoid arthritis, spondyloarthritis, and periprosthetic infections (30-32). In our cohort, CAR was significantly higher in patients with AIS and positively correlated with the Cobb angle, which suggests it may reflect a chronic low-grade inflammatory state relevant to curve severity. Given that CAR combines CRP and albumin, it may more accurately represent systemic inflammation than single markers. Whether CAR could serve as a prognostic biomarker for AIS progression warrants further prospective validation.

Although our study demonstrated significant associations between inflammatory indices and curve severity, the etiology of AIS is multifactorial, and our results cannot establish causality. Potential genetic, endocrine, and neuromuscular contributions may interact with systemic inflammation, an issue that requires further study. These markers may assist in early risk stratification and closer monitoring of patients at a higher risk of curve progression. If validated prospectively, they could also help stratify follow-up intensity, for example, by guiding more frequent radiographic and clinical monitoring in patients with elevated CAR or NLR at baseline. However, routine testing cannot yet be recommended in daily clinical practice. Therefore, we do not advocate universal laboratory screening for all patients with AIS, as this would increase costs and patient burden without proven benefit. Instead, such indices may be useful adjuncts in research or in selected patients at high risk of progression.

The study has several limitations. First, its retrospective design limits causal inference and is prone to residual confounding despite strict exclusion criteria. Nutritional status, vitamin D levels, genetic predisposition, and subclinical infections were not systematically assessed and may have influenced inflammatory parameters. Moreover, emerging machine-learning models integrating clinical, radiological, and biochemical variables show promise in predicting curve progression, which underscores the need for prospective studies incorporating broader risk factors (33). Second, the absence of longitudinal follow-up or treatment outcomes restricts interpretation regarding prognostic utility. Third, although controls underwent full-spine radiography to confirm the absence of scoliosis, selection bias cannot be completely excluded. Finally, laboratory values were not standardized across centers, and inter-laboratory variability may have affected measurements.

In conclusion, this study is the first to evaluate CAR in AIS within a large cohort including a control group with a Cobb angle of 0°. These findings suggest that simple and low-cost markers such as NLR and CAR may provide supportive information at diagnosis. However, their role in prognosis and clinical decision-making requires confirmation in prospective studies, and routine universal laboratory screening is not currently justified.

*Data are presented as mean (standard deviation).
